# Combination of Treatments With or Without Surgery in Localized Provoked Vulvodynia: Outcomes After Three Years of Follow-Up

**DOI:** 10.1089/biores.2018.0044

**Published:** 2019-03-08

**Authors:** Anu Pauliina Aalto, Heini Huhtala, Johanna Mäenpää, Synnöve Staff

**Affiliations:** ^1^ Faculty of Medicine and Life Sciences, University of Tampere, Tampere, Finland.; ^2^ Department of Obstetrics and Gynecology, Kanta-Häme Central Hospital, Hämeenlinna, Finland.; ^3^ Faculty of Social Sciences, University of Tampere, Tampere, Finland.; ^4^ Department of Obstetrics and Gynecology, Tampere University Hospital, Tampere, Finland.

**Keywords:** quality of life, RAND-36, vestibulectomy, vulvodynia, vulvodynia treatment

## Abstract

Most vulvodynia patients receive combinations of several treatment modalities for their chronic painful condition. If conservative treatments fail, vestibulectomy is considered to be the ultimate treatment option for localized provoked vulvodynia (LPV). The aim of this descriptive study was to analyze relief of pain, quality of life (QoL), and complications associated with combining surgery with conservative treatments among LPV patients, both in short term and after 3 years of follow-up.

The study population consisted of a retrospective patient cohort of surgically (*n* = 16) and only conservatively (*n* = 50) treated LPV patients. QoL data were assessed by a validated questionnaire (RAND-36). Data were collected by reviewing patient records and by aid of postal questionnaires. Efficacy of treatments in relief of pain was measured by numerical rating scale (NRS). Two months after surgery, the NRS scores assessed by a physician were lower in the surgery group than in patients treated only conservatively (*p* = 0.008). However, after a median of 36 months of follow-up, self-reported NRS scores and QoL showed no difference between the two patient cohorts. Complication rate after vestibulectomy was 18.8%. The findings suggest that combining surgery with conservative treatments may result in a more effective short-term reduction of pain. However, the effect seemed to be only temporary, as no long-term benefit was achieved.

## Introduction

Vulvodynia is a chronic pain syndrome of unknown etiology affecting 7–8% of women in population-based epidemiological studies.^1,2^ Vulvodynia is usually described as burning, stabbing, itching, stinging, and feeling of irritation. The 2015 Consensus and Terminology and Classification of Persistent Vulvar Pain and Vulvodynia^3^ divides vulvar pain into two categories. The first category includes vulvar pain that is caused by a specific clearly identifiable disorder (e.g., pain caused by genital herpes). The second category includes vulvar pain that is at least 3 months in duration and cannot be clearly identified or linked to a specific cause. However, it may have potential associated factors. The descriptors of the pain are location (local, generalized, and mixed), type (provoked, spontaneous, or mixed), onset (primary and secondary), and temporal pattern (intermittent, persistent, constant, immediate, and delayed). Symptoms can overlap and co-occur. Vulvodynia may be associated with a history of yeast infection, hormonal factors, genetic factors, pelvic floor dysfunction, and psychological factors.^3^

The most common clinical subtype of vulvar pain in premenopausal women is localized provoked vulvodynia (LPV).^4^ LPV is also considered to be the most common form of sexual pain in women <30 years of age.^5^ Chronic pain is known to have a negative impact on a woman's quality of life (QoL).^6,7^

Different medical treatment modalities for LPV consist of local, topical, or oral medications. Patients treated by a multidisciplinary team are usually offered physiotherapy (including transcutaneous electrical nerve stimulation), sexual counseling and therapy, and psychotherapy. Although a multidisciplinary approach to LPV is recommended,^8,9^ it is actually not evidence based.^10^ Surgery (vestibulectomy) for LPV is recommended as the ultimate treatment option, if conservative treatments fail or are insufficient in terms of pain reduction.

Based on studies concerning surgical treatment for LPV, reported success rates vary between 60% and 90%,^11^ even though the comparison of different studies is difficult as the term “success,” the surgical technique used and the length of follow-up show considerable variation.^11^ There is no definitive consensus as to which surgical technique is the superior one. In a review by Tommola et al.,^12^ which was based on 33 studies on surgical treatment for LPV (or vulvar vestibulitis), it was concluded that the experience of individual surgeons plays an important role, and that the aim of surgery should be to remove all painful tissues while avoiding unnecessary risks. The review also found surgery to be effective and safe.^12^

Most studies on surgical treatment for LPV lack randomization and/or controls. One of the few randomized controlled studies on vestibulectomy is that by Bergeron et al.,^13^ which showed that vestibulectomy was more successful than surface electromyographic feedback and group cognitive-behavioral therapy in pain reduction. As the authors stated, there is a concern in interpreting these results, due to a higher pretreatment drop-out rate in the vestibulectomy group.^13^ However, the psychological and sexual functions remained equally positive in all three groups after 6 months of follow-up. Another study that included randomization to the surgical (behavioral treatment and surgery) and nonsurgical (behavioral treatment only) groups, by Weijmar Schultz et al.,^14^ found no difference in the outcomes between these two treatment modalities after a mean of 2.5–3 years of follow-up. In the review of Goldstein et al.,^11^ surgery was recommended for LPV after failure of conservative treatments (level B evidence).

In previous studies concerning surgery for LPV, the measured outcomes have varied. At least pain reduction,^13,15^ dyspareunia,^13,16^ sexual functioning,^13^ psychological distress,^15^ and patient satisfaction^16^ have been measured using questionnaires; moreover, findings of physical examination and self-reported symptoms have also been reported. Psychological well-being,^17^ quality of sexual life,^17^ and sexual and partnership satisfaction have all been reported to improve^18^ after vestibulectomy.

The aim of this study was to evaluate the safety and effectiveness of LPV treatments with or without surgery in both short and long terms. Pain was measured by numerical rating scale (NRS) assessed by both a physician and the patient. QoL was evaluated after a combination of treatments with or without surgery, using a questionnaire (RAND-36) validated in the Finnish population.

## Materials and Methods

**FIG. 1.**
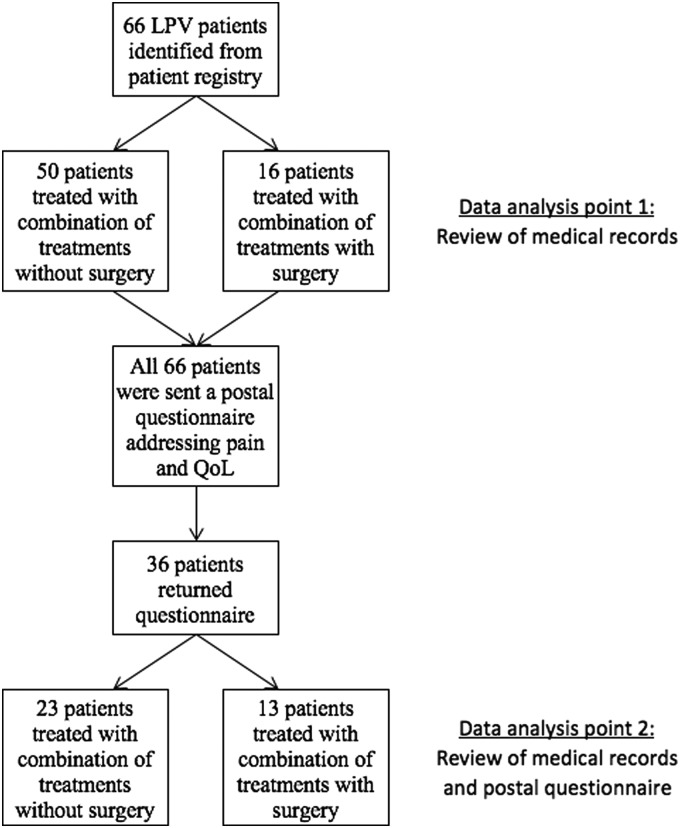
Patient flow chart.

This retrospective cohort study on LPV patients was carried out at the Department of Obstetrics and Gynecology of Tampere University Hospital (TAUH), Tampere, Finland. All at least 18-year-old women diagnosed with vulvodynia at TAUH from January 2003 to May 2016 were screened for the study. Potential vulvodynia patients were identified from the hospital records (computer database) by using the appropriate ICD-10 codes: N90.9 (noninflammatory disorder of vulva and perineum, unspecified); N90.8 (other specified noninflammatory disorders of vulva and perineum); N94.1 (dyspareunia); and N94.2 (vaginismus). Only LPV patients who fulfilled the strict criteria by Friedrich,^19^ or severe pain on vestibular touch or attempted vaginal entry, and tenderness on localized pressure within the vulvar vestibule, were considered eligible (*n* = 66). Among these eligible patients, 16 patients operated on for LPV (vestibulectomy) were identified. Patients with generalized or continuous vulvar pain were excluded. Other exclusion criteria included malignant tumors of vulva and ongoing inflammatory or dermatological diseases of vulva. The flow chart of study patients is shown in Figure 1.

Information on parity, menopausal status, age, different treatment modalities, and complications after surgery was collected from the hospital's medical records. The baseline pain before any treatments for LPV was assessed by a physician with a cotton swab test and rated on an NRS from 0 to 10. If the rating was not found in the patient record, the information was reported as “no data.” As a part of the treatment protocol, every patient had a checkup appointment at 2 months after the surgical treatment with the operating surgeon. Patients treated with conservative methods only were assessed by a physician usually after 2 or 3 months after commencing the treatments. The conservative treatment modalities used for LPV are described in Table 1.

**Table 1.** Demographic Data and Treatments Given to Localized Provoked Vulvodynia Patients

	Data analysis point 1 (Fig. 1). Review of medical records				Data analysis point 2 (Fig. 1). Review of medical records and postal questionnaire			
	All LPV patients	Combination of treatments without surgery	Combination of treatments with surgery	*p*^a^	All LPV patients	Combination of treatments without surgery	Combination of treatments with surgery	*p*^a^
Number of patients	66	50	16	N/A	36	23	13	N/A
Age, median (IQR)	28 (25–33)	27 (24–32.3)	30.5 (26.5–38.3)	0.048	28.5 (25–32)	27 (24–29)	29 (26.5–33)	0.06
Nulliparous, % (*n*)	95.5 (63)	94 (47)	100 (16)	0.32	86 (31)	82.6 (19)	92.3 (12)	0.48
Premenopausal, % (*n*)	98.5 (65)	100 (50)	93.8 (15)	0.08	100 (36)	100 (23)	100 (13)	1.00
NRS before treatments, asked from patients at the time of the cotton-swab test	9 (7.25–9), n.d. *n* = 22	9 (7–9), n.d. *n* = 18	9 (8–9.5), n.d. *n* = 4	0.11	9 (7–9), n.d. = 9	8 (7–9), n.d. *n* = 7	9 (8–10), n.d. = 2	0.014
NRS after treatments, asked from patients at the time of the cotton-swab test	5 (2–8), n.d. *n* = 24	7 (4–8), n.d. *n* = 19	2 (2–4), n.d. *n* = 5	0.008	5 (2–7), n.d. *n* = 10	7 (4.5–8), n.d. = 7	2 (2–4), n.d. = 3	0.005
Self-reported NRS before treatments in the postal questionnaire	N/A	N/A	N/A	N/A	8 (8–9)	8 (7–9)	8 (8–9)	0.66
Self-reported NRS after follow-up in the postal questionnaire	N/A	N/A	N/A	N/A	3 (2–5.75)	4 (3–6)	2 (2–5)	0.18
Treatments received by LPV patients								
Local treatments,^b^ % (*n*)	100 (66)	100 (50)	100 (16)	1.00	100 (36)	100 (23)	100.0 (13)	1.00
TCA or anticonvulsant^c^	15.2 (10)	12.0 (6)	25.0 (4)	0.21	16.7 (6)	13.0 (3)	23.1 (3)	0.35
Physiotherapy (including TENS)	90.9 (60)	92.0 (46)	87.5 (14)	0.59	88.9 (32)	91.3 (21)	84.6 (11)	0.46
Sexual counseling by a trained nurse	75.8 (50)	80.0 (40)	62.5 (10)	0.16	77.8 (28)	87.0 (20)	61.5 (8)	**0.03**
Topical treatments^d^	22.7 (15)	18.0 (9)	37.5 (6)	0.11	19.4 (7)	8.7 (2)	38.5 (5)	0.050
Local injections to the painful site^e^	16.7 (11)	16.0 (8)	18.8 (3)	0.80	11.1 (4)	8.7 (2)	15.4 (2)	0.76

^a^ *p*-value between surgical and nonsurgical groups.

^b^ Lidocaine gel to the painful area in vulva 30 min before intercourse or gabapentin 6% cream applied twice a day to the painful area for 6–8 weeks.

^c^ Amitriptyline 10–40 mg most commonly used TCA or pregabalin 150–300 mg.

^d^ Podophyllotoxin (5 mg/mL) applied locally to tender points of vestibulum after 5% acetic acid application. Treated area was covered with a mild estrogen cream and covered with gauze pads until the next day.

^e^ 2–4 mL of betametasone and long acting anesthetic agent (bupivacaine), both 50% and 50%, injected submucuously to the painful site.

IQR, interquartile range; LPV, localized provoked vulvodynia; N/A, not applicable; n.d., no data; NRS, numerical rating scale; TCA, tricyclic antidepressant; TENS, transcutaneous electrical nerve stimulation.

The surgical technique used was the modified posterior vestibulectomy described by Tommola et al.,^16^ with the aim to surgically remove the painful vulvar area. The operations were performed under general anesthesia, and all operations were carried out by three senior gynecological surgeons. First, 0.01% lidocain cum adrenalin solution was injected into the vulvar vestibulum for bleeding control and prevention of postoperative pain. To excise vestibular mucosa, 2-mm deep incisions using electrocautery were made from 10 to 2 o'clock in the posterior vulvar vestibulum to a width of ∼1–2 cm. The inner incision was made just inside the hymenal ring, and the outer margin followed the Hart's line. The vaginal mucosa was liberated from underlying tissue and subsequently opposed to distal vulvar margin with absorbable sutures without tension.

A seven-page postal questionnaire on demographic data, self-reported pain, and RAND-36 was sent to the 66 eligible LPV patients. The questionnaire was resent to the patients who did not return the questionnaire within 2 months after the first mailing.

The validated Finnish version of the RAND-36-item health survey includes eight multi-item dimensions: general health, physical functioning, mental health, social functioning, vitality, pain, and physical and emotional role functioning.^20,21^

Participants of the study were moreover asked to assess vulvar pain intensity upon touch on the NRS before and after treatments. NRS was used to quantify the intensity of vulvar pain by rating the pain using a 0-to-10 scale, where 0 indicates “no pain” and 10 indicates “the worst pain imaginable.”

The study protocol was approved by TAUH Ethical Committee (5APR2016, Identification Code R16053), and a written informed consent was obtained from the patients participating in this study.

Version 23 of IBM SPSS statistics software was used in statistical analyses (IBM SPSS Statistics for Windows, Version 23.0. IBM Corp. 2015. Armonk, NY). Mann–Whitney *U*-test was used for statistical comparisons. A probability value of *p* < 0.05 was considered as statistically significant.

## Results

Thirty-six patients (55%) returned the questionnaire during the study period (August 2016–November 2016). Twenty-eight patients returned the questionnaire after the first mailing and eight patients after the second mailing. The patient flow chart is shown in Figure 1. The response rate to postal questionnaires in the nonsurgical group was 46.0% and that in the surgical group was 81.3% (*p* = 0.020). Demographic data and pain before and after the treatments are shown in Table 1. At the data analysis point 1 (2 months after commencing the treatments), the surgical and nonsurgical groups differed significantly in age (*p* = 0.048). The median follow-up time at the data analysis point 2 was 36 months (interquartile range [IQR] = 24–36). The most frequent (received by >50% of the patients) combination of conservative treatments consisted of local treatments (lidocaine and/or gabapentin), physiotherapy, and sexual counseling in both patient cohorts. The treatment modalities used for both patient groups are summarized in Table 1. At the data analysis point 1, the nonsurgical and surgical treatment groups did not differ with respect to any treatment modality. However, at the data analysis point 2, the two treatment groups differed with respect to the frequency of sexual counseling (Table 1; *p* = 0.03).

At data analysis point 1, median pretreatment NRS scores were similar between nonsurgical (i.e., combination of treatments without surgery) and surgical groups (median NRS scores 9 in both groups, *p* = 0.11, Table 1). Median post-treatment NRS score assessed by a physician in different treatment groups was 7 and 2, respectively (*p* = 0.008). After median of 36 months of follow-up, self-reported NRS scores before or after treatments did not differ significantly between the groups (*p* = 0.66 and *p* = 0.18, respectively, Table 1). At data analysis point 2, we also compared medical record-derived data assessed by a physician. Physician-assessed NRS score before treatment in the nonsurgical group was 8 and that in the surgical group was 9 (*p* = 0.014). Similarly, post-treatment NRS score assessed by a physician was 7 and 2, respectively (*p* = 0.005). Among the LPV patients who did not respond to postal questionnaires (*n* = 30), the median pretreatment NRS score collected from the patient records was 9 (IQR = 8–9.5, missing data *n* = 13), and the median 2-month post-treatment NRS score was 5 (IQR = 2.25–8, missing data *n* = 14). When nonresponders were compared with all LPV patients who returned the questionnaire (data analysis point 2), the pre- and post-treatment NRS scores derived from the medical records were similar (*p* = 0.291, *p* = 0.592, respectively).

**FIG. 2.**
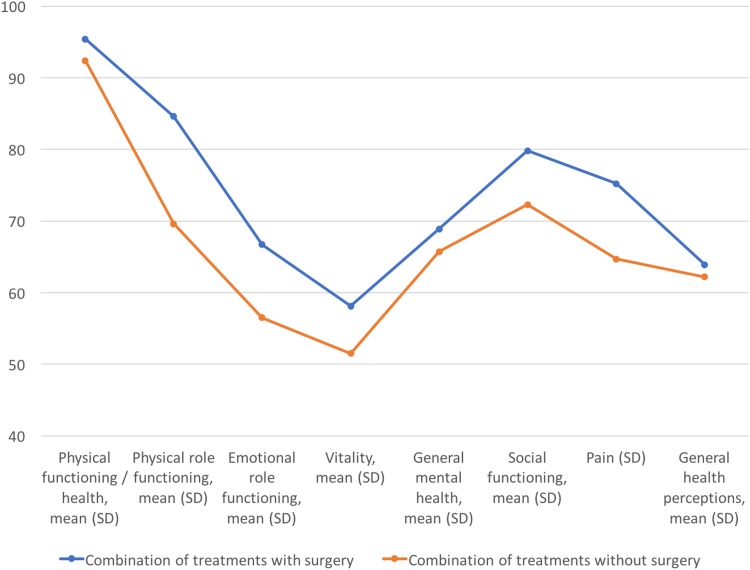
Quality of life of LPV patients measured with RAND-36. LPV, localized provoked vulvodynia.

The QoL after a median of 36 months of follow-up after treatments did not differ significantly between the surgical and nonsurgical groups in any of the eight multi-item dimensions (Table 2 and Fig. 2).

**Table 2.** Quality of Life After Follow-Up in Different RAND-36 Dimensions

	Combination of treatments with surgery	Combination of treatments without surgery	*p*^a^
Physical functioning/health, mean (SD)	95.4 (15.20)	92.4 (14.45)	0.243
Physical role functioning, mean (SD)	84.6 (33.13)	69.6 (43.92)	0.278
Emotional role functioning, mean (SD)	66.7 (40.82)	56.5 (46.53)	0.498
Vitality, mean (SD)	58.1 (16.65)	51.5 (23.95)	0.518
General mental health, mean (SD)	68.9 (22.87)	65.7 (21.77)	0.416
Social functioning, mean (SD)	79.8 (19.46)	72.3 (27.94)	0.485
Pain, (SD)	75.2 (26.76)	64.7 (24.50)	0.144
General health perceptions, mean (SD)	63.9 (21.03)	62.2 (23.88)	0.974

^a^ *p*-value between surgical and nonsurgical treatment groups.

SD, standard deviation.

Out of 16 patients operated on, 3 had complications after surgery, resulting in a complication rate of 18.8%. One patient had heavy postoperative pain and was readmitted to hospital on the third postoperative day. Two months after surgery, the patient was still suffering from pain, whereas after 1 year of follow-up the pain in the vulvar area was “transformed into a neuropathic pain,” and the patient was treated with peroral gabapentin, which resulted in sufficient pain relief. Another patient was readmitted after 7 days of surgery, because of a partial wound dehiscence. The wound was reported to have healed completely at the 2-month follow-up visit. The third patient suffered from severe pain right after surgery, and had to stay overnight at the hospital. At 2-month follow-up, the pain score was assessed as “0” by the operating physician.

## Discussion

We describe here a retrospective cohort of 66 LPV patients treated at our institution. We evaluated short-term surgical complications, pain, and QoL of nonsurgically and surgically treated patients first after 2 months and then after a median of 36 months of follow-up. QoL after 36 months did not differ when comparing the surgically and only conservatively treated groups in any of the eight QoL dimensions of validated RAND-36 questionnaire. Addition of surgery to the conservative treatments resulted in lower NRS scores measured by a physician 2 months after surgery. However, there was no difference in self-reported NRS pain scores measured after the longer follow-up.

Vestibulectomy seems to be a safe treatment modality for LPV with an acceptable complication rate. This is in line with the previous review by Tommola et al. concerning surgery for LPV.^12^ In this study, surgery was associated with better short-term outcomes in terms of pain after 2 months of surgery. Previously, it has been shown that median pain measured with VAS decreases from 8 to 2 in surgically treated patients,^18^ which is of a magnitude similar to our results. However, assessment of pain at the checkup visits shortly or at any time point after surgery by the attending surgeon is not blinded and certainly at risk of many types of bias. This bias may also explain the differences shown here between NRS values obtained from medical records and those reported by patients themselves. In a randomized study,^14^ surgical intervention added to behavioral approach had outcome similar to behavioral approach after 2.5–3 years of follow-up. A similar outcome was found among patients given an opportunity to choose between surgery and no surgery. Although the sample size in the study was small (*n* = 14 in the randomized part of the study), it being a randomized study strengthens the perception that individual tailoring of treatment is one of the key factors in a successful treatment for LPV.

We report here QoL data obtained with a validated questionnaire among surgically and nonsurgically treated vulvodynia patients. There was no difference in QoL between these two patient groups after a median of 3 years of follow-up. Previously, Bohm-Starke and Rylander have reported that vestibulectomy improves QoL measured by VAS from median 0.5 to 6.5, during a median 41 months of follow-up.^17^ However, another long-term follow-up study on LPV patients treated conservatively versus treated surgically failed to show any difference in long-term well-being between the treatment groups.^18^ Even if there are previous valuable reports on QoL and overall well-being among vestibulectomy patients,^17,18^ to our knowledge this is the first report using a validated QoL questionnaire when assessing QoL among vestibulectomy patients.

There are some limitations to our study. The study is a retrospective nonrandomized cross-sectional study. An ideal study setting would have been a comparison between only surgically and nonsurgically treated patients preferably as a randomized controlled study. A confounding factor is that the study patients in the surgical group had also received various conservative treatments before surgery, that is, the comparison between the groups is in fact a comparison between combination of treatments with and without surgery. Because both groups received various conservative treatment modalities it is not possible to conclude fully the effectiveness of surgery. However, this setting is clinically unavoidable since vestibulectomy is the treatment modality offered to patients only after failure of all noninvasive treatments. The fact that patients were asked to report pain retrospectively after a median of 36 months after treatments contains also a risk of bias. However, the median follow-up time after treatments did not differ significantly between surgically and nonsurgically treated patients (*p* = 0.35) and QoL measured corresponded to present moment (i.e., the time of questionnaire). A longitudinal QoL evaluation, done before and after treatments, would have been of additional value.

Although the total number of patients is relatively low, with the surgically treated group being even smaller, it reflects the fact that vulvodynia is a rather rare condition. The response rate after follow-up was only satisfactory, 55%. The response rate to postal questionnaires of surgically treated patients was higher and this may lead to a false accentuation of positive effect of the intervention, that is, to a type I error. However, pain rated on the NRS and QoL did not differ significantly between the groups after the longer follow-up. The amount of missing data was unfortunately also quite high and this may cause bias. The study patients had received slightly different conservative treatment entities before surgery or during the treatment period that might have an effect on outcome, too.

## Conclusion

Measuring QoL with a validated questionnaire in the Finnish population can be considered as strength of the study. Bearing in mind the limitations, as discussed earlier, we conclude that even if surgery seems to be associated with more effective pain management in the short term, it showed no additional benefit with respect to QoL or pain after extended follow-up. In contrast, it may be concluded that performing vestibulectomy after conservative treatments is safe and does not seem to be harmful. However, long-term patient-reported outcomes in terms of QoL and pain after surgery do not seem to differ from those achieved conservatively. Considering recent evidence of a strong placebo effect concerning medical treatments,^22^ prospective sufficiently powered controlled trials are truly warranted.

## Abbreviations Used

IQR: interquartile range
LPV: local provoked vulvodynia
N/A: not applicable
n.d.: no data
NRS: numerical rating scale
QoL: quality of life
TAUH: Tampere University Hospital
TCA: tricyclic antidepressant
TENS: transcutaneous electrical nerve stimulation
VAS: visual analogue scale
